# Matrons, and Nurse Agitators

**Published:** 1918-09-14

**Authors:** 


### THE EDITOR'S LETTER-BOX.

[Correspondence on all subjects is invited, but we cannot in any way be responsible for the opinions expressed by our correspondents, who must give their name and address as a guarantee of good faith, but not necessarily for publication. Correspondents are reminded that brevity of style and conciseness of statement greatly facilitate early insertion.]

### Matrons, and Nurse Agitators.

*To the Editor of* The Hospital.

Sir,—In your issue for July 13 there was an article relating to the protest being raised by some of our Australian sisters against the absolute preponderance of the matron's opinion of nurses under her control. I, together with several of my colleagues, heartily agree with them, and think that a similar movement ought to be commenced here in England. Over and over again we have evidence of how a matron can make or mar a girl's career. Unfortunately, we still have a few sisters and matrons who seem incapable of being absolutely impartial to personal likes or dislikes when reporting on a nurse's conduct and work in the wards. It is well known that if a matron takes a dislike to a nurse she may as well "throw up the sponge," as she will never be really happy. In my two and a half years in this training-school I have seen nurses who are good, conscientious workers, and will make excellent administrators in the future, led a "dog's life," simply because they are what "Ierne" calls agitators. Such are the ones who fight for the others, and get themselves heartily disliked by the "powers that be." Why should this be so? Why cannot nurses be free to express their opinions or complaints freely and openly? We may not do so simply because there is always that dread at the back of one's mind that matron may not give us a testimonial at the end, and so blight our careers. Another thing: I do not know if there are many probationers similarly situated, but it is the custom in this training-school never to give more than "good" on our certificates for any subject. It is common knowledge that in most training-schools "very good," and in some cases "excellent" is given for conduct, etc. How shall we fare when applying for posts in the future? What is our "best" certificate will probably only appear very ordinary to another matron, whilst we may be equally as good as another nurse who has "excellent." What says "Ierne"?

A Third Year Pro.

[We have good reason to believe there are 5,000 or more nurses in training who are third-year probationers. Our correspondent may be unfortunately circumstanced. In any case we hope to have letters on the points raised from some of the inarticulate 4,999 who have not yet sent us their views and experience. Full discussion will do real good providing it is realised that the aim of each writer should be to lead her sister nurses to ponder and fully consider each question debated from every point of view. The best of matrons is the best of friends and advisers for her probationers and nurses. This the keenest and fairest probationer who wishes to become a successful nurse will agree. But is it not possible that some there are who in moments of irritation may welcome discussion and use it in a seldom used meaning of the word, *i.e.*, "to consume with enjoyment" something or somebody?—Ed. The Hospital.]

A Probationer Nurse who is Afraid to Sign her Name:—The cause shall be taken up if you make an appointment at once to see the Editor confidentially at 13 Porchester Square, London, W. 2, on the earliest day possible for your friends and yourself.—Ed. The Hospital.

